# A New Strategy for Rapidly Screening Natural Inhibitors Targeting the PCSK9/LDLR Interaction In Vitro

**DOI:** 10.3390/molecules23092397

**Published:** 2018-09-19

**Authors:** Li Li, Chen Shen, Ya-Xuan Huang, Ya-Nan Li, Xiu-Feng Liu, Xu-Ming Liu, Ji-Hua Liu

**Affiliations:** 1 Jiangsu Key Laboratory of TCM Evaluation and Translational Research, School of Traditional Chinese Pharmacy, China Pharmaceutical University, Nanjing 211198, China; lili_flying@126.com (L.L.); pharma_sc@163.com (C.S.); 1722020478@stu.cpu.edu.cn (Y.-X.H.); liyanan_cpu@163.com (Y.-N.L.); xf.liu@cpu.edu.cn (X.-F.L.); 2 School of Life Science and Technology, China Pharmaceutical University, Nanjing 211198, China; 3 State Key Laboratory of Natural Medicines, China Pharmaceutical University, Nanjing 211198, China

**Keywords:** PCSK9, LDLR, interaction, natural products, hypercholesterolemia

## Abstract

The interaction between proprotein convertase subtilisin/kexin type 9 (PCSK9) and the low-density lipoprotein receptor (LDLR) is a promising target for the treatment of hyperc-holesterolemia. In this study, a new method based on competitive affinity and tag detection was developed, which aimed to evaluate potent natural inhibitors preventing the interaction of PCSK9/LDLR directly. Herein, natural compounds with efficacy in the treatment of hypercholesterolemia were chosen to investigate their inhibitory activities on the PCSK9/LDLR interaction. Two of them, polydatin (**1**) and tetrahydroxydiphenylethylene-2-*O*-glucoside (**2**), were identified as potential inhibitors for the PCSK9/LDLR interaction and were proven to prevent PCSK9-mediated LDLR degradation in HepG2 cells. The results suggested that this strategy could be applied for evaluating potential bioactive compounds inhibiting the interaction of PCSK9/LDLR and this strategy could accelerate the discovery of new drug candidates for the treatment of PCSK9-mediated hypercholesterolemia.

## 1. Introduction

Hyperlipidemia is a metabolic disorder involving increased levels of lipids and/or lipid proteins in the blood [1], in which high cholesterol levels in the plasma (hypercholesterolemia) have been identified as a major cause of arteriosclerosis, heart disease and stroke [2], leading to prominent adult mortality worldwide. The low-density lipoprotein receptors (LDLR) can capture and transport dissociative low-density lipoprotein receptors-cholesterol (LDL-C) into a cell [3], after which the LDLR can return back to the cell surface, achieving cholesterol control [4]. However, proprotein convertase subtilisin/kexin type 9 (PCSK9), which is overexpressed in patients with familial hypercholesterolemia, competitively binds to the LDLR and inhibits the rearrangement of the LDLRs recycled to the plasma membrane, leading to high levels of LDL-C in the plasma [5,6]. Multiple clinical studies have shown that antibodies that impede the PCSK9/LDLR protein–protein interaction could significantly decrease the circulating LDL-C levels [3]. Thus, the PCSK9/LDLR interaction is currently deemed as an important target for the treatment of hypercholesterolemia. Although two antibody drugs have been approved as PCSK9 inhibitors, orally available, small molecule inhibitors of PCSK9 would be highly desirable agents for alternative therapy due to their lower cost and ease of administration [7,8,9].

Herbal medicines are widely used for the treatment of hypercholesterolemia, and some natural products have been reported to regulate the transcription and expression of PCSK9 because of their cholesterol-lowering effects [10,11,12,13]. However, only a few natural products in either extracts or individual chemicals have been explored for interrupting the PCSK9/LDLR interaction directly.

**Figure 1:**
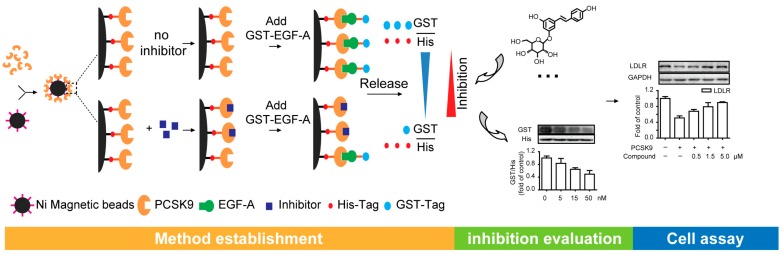
Strategy for evaluating the inhibition of natural products on the proprotein convertase subtilisin/kexin type 9 (PCSK9)/low-density lipoprotein receptor (LDLR) interaction. PCSK9 with a hexahistidine-tag was immobilized on Ni magnetic beads. The epidermal growth factor precursor homology domain A (EGF-A), a domain of the LDLR contributing to PCSK9 binding, was chosen for simulating the function of the LDLR with the addition of a glutathione S-transferase (GST)-tag. When the inhibitors were introduced, the PCSK9/EGF-A interaction was interrupted, and the ratios of the tags (GST/His) represented the inhibitory activities. The inhibition by the natural inhibitor was screened in this method and validated by cell assays.

Several valuable methods have been reported for the evaluation of the protein–protein interaction, such as co-immunoprecipitation [14], alphascreen technology [15], surface plasmon resonance and ELISA [16], while the costs of such methods need to be taken into consideration for large-scale screening. Thus, we aimed to establish an integrated method that could be used to evaluate the activities of natural products on the PCSK9/LDLR interaction conveniently and economically, which maintain the advantages of the previous methods and can be used for preliminary screening. A schematic diagram of our approach is illustrated in Figure 1. PCSK9 with a hexahistidine-tag was primarily immobilized on magnetic beads (MBs). The epidermal growth factor precursor homology domain A (EGF-A) of the LDLR is critical for PCSK9 binding [17,18], and the GST-tagged EGF-A has been proven to bind to PCSK9 [19]. So, we chose PCSK9 (His-tagged) and EGF-A (glutathione S-transferase-tagged, GST-tagged) to simulate the PCSK9/LDLR interaction in this study. When the inhibitors were introduced, we hypothesized that the protein–protein interaction would be interrupted, presenting changes in the proportion of EGF-A binding to PCSK9. The ratio of the tags (GST/His) could then be used for evaluating the inhibitory activities. Natural products with cholesterol-lowering effects, such as polydatin (**1**) [10,20], tetrahydroxy- diphenylethylene-2-*O*-glucoside (**2**) [21] and crocin I (**3**) [22], were selected as the candidates for screening the potential natural inhibitors targeting the PCSK9/LDLR interaction. A cell assay was also conducted for further verification. The present strategy is expected to accelerate the natural product-based drug discovery for the treatment of hypercholesterolemia.

## 2. Results and Discussions

### 2.1. Expression and Purification of His-PCSK9 and GST-EGF-A

**Figure 2:**
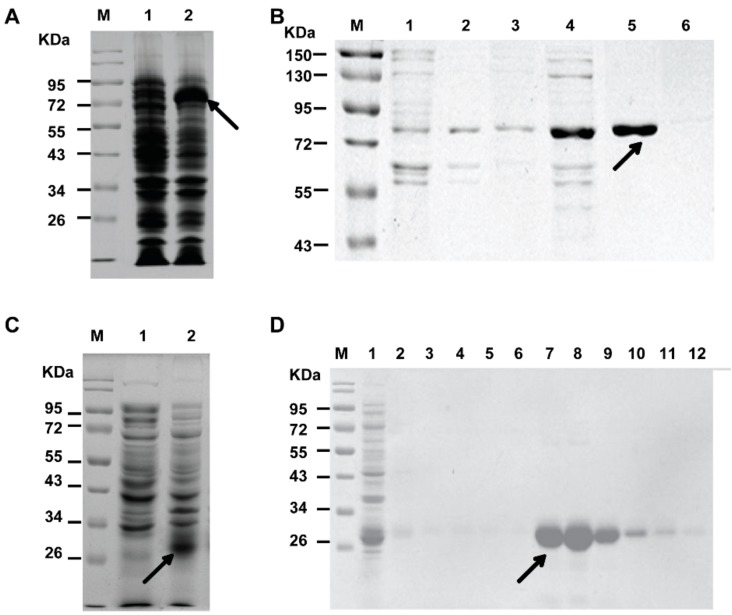
Expression and purification of His-PCSK9 and GST-EGF-A. (**A**) Expression of His-PCSK9 (Lane M: prestained protein marker; Lane 1: cell lysate before induction with isopropylthio-β-d-galactopyranoside (IPTG); Lane 2: cell lysate after 24 h of expression); (**B**) Purification of His-PCSK9 (Lane M: pre-stained protein marker; Lane 1–6: elution by buffer with 2 mM, 5 mM, 10 mM, 25 mM, 50 mM, and 250 mM imidazole, respectively); (**C**) Expression of GST-EGF-A (Lane M: pre-stained protein marker; Lane 1: cell lysate before induction with IPTG; Lane 2: cell lysate after 24 h of expression); (**D**) Purification of GST-EGF-A (Lane M: prestained protein marker; Lane 1–6: washing with 6-column volumes of buffer in turn; Lane 7–12: eluting with 6-column volumes of buffer containing glutathione in turn).

The best-characterized function of secreted PCSK9 is to target the LDLR and it induces the degradation of the LDLR. The catalytic subunit of PCSK9 contains a site that binds to the epidermal growth factor-A (EGF-A) domain of the LDLR at the hepatocyte cell surface, ultimately leading to LDLR degradation [23,24,25]. Moreover, the C-terminal domain of PCSK9 could interact with the LDLR at endosomal pH, which could lead to receptor degradation [26]. So, the catalytic domain and C-terminal domain of human PCSK9 were chosen as sites for expression. Herein, human PCSK9 (152-692) was expressed in *Escherichia coli* BL21 (DE3) and purified with a hexahistidine-tag at the N-terminus. As shown in Figure 2A, the protein (His-PCSK9) was successfully expressed at the target molecular weight (~72 kD). The purification procedure was optimized and buffers with 50 mM imidazole, which was selected for eluting and collecting His-PCSK9 (Figure 2B).

Due to the important role of the EGF-A in the PCSK9/LDLR interaction mentioned above, we aimed to express and purify the EGF-A domain of the LDLR for the exploration of the protein–protein interaction. The expression results were presented in Figure 2C. The EGF-A was successfully expressed by adding a GST-tag at the N-terminus (GST-EGF-A) for subsequent purification according to the previous literature [19]. In the GST-tag purification process, volumes of washing and elution were critical factors for the purification of the target protein. As shown in Figure 2D, washing with 5-column volumes of wash buffer and then eluting with 6-column volumes of elution buffer were proven to be optimal the optimal procedures.

### 2.2. Establishment of the Method for Evaluating the Inhibitory Activities on PCSK9/LDLR Interaction

PCSK9 could immobilize on magnetic beads (MBs) which were easy to adsorb and to use to separate the potential ligands rapidly. The EGF-A, the active binding domain on the LDLR, was chosen for simulating the competitive binding characteristics of the LDLR. When the inhibitors were introduced, we speculated that the interaction between PCSK9 (6 × His-tagged) and EGF-A (GST-tagged) would be interrupted, leading to a decrease of the ratio of the tags (GST/His) on the MBs 

**Figure 3:**
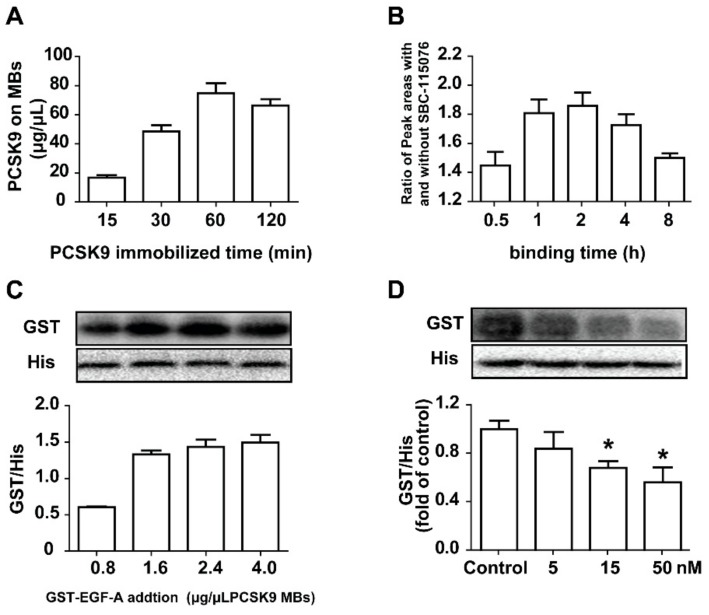
Establishment of the method for evaluating the PCSK9/LDLR interaction. The effects of the immobilized time of the PCSK9-MBs (**A**); the binding time between the ligands and the PCSK9-MBs (**B**) and the amounts of GST-EGF-A (**C**) on the binding assay were investigated; (**D**) The method established was verified by mixing GST-EGF-A (2.4 μg/μL PCSK9-MBs) and the PCSK9-MBs in presence of positive compound SBC-115076 with different concentrations (5, 15, and 50 nM), and the GST/His ratios were monitored by western blot. The control group was conducted without the addition of SBC-115076. The values are the mean ± SEM deviation of the three independent experiments. * *p* < 0.05; ** *p* < 0.01, compared with the control group.

The Ni^2+^ of the MBs can be chelated to the hexahistidine tag of PCSK9, and the PCSK9-coated MBs (PCSK9-MBs) could be formed. The incubation time is important for such an immobilized process. Incubation times between 15 and 120 min were tested, and 60 min was confirmed to be enough time for PCSK9 immobilization (Figure 3A). By emulating the interaction between PCSK9 and the EGF-A of the LDLR in the cells, we speculated that the PCSK9-MBs could bind to GST-EGF-A in vitro. Considering the stability and feasibility of the competitive adsorption process, adding excess GST-EGF-A was necessary. Different ratios of EGF-A/PCSK9 were mixed, and the ratio at 2.4 μg EGF-A/μL MBs was proven to be optimal (Figure 3C). Long-time incubation may cause the devitalization of the enzymes, resulting in lower binding degrees. To screen for the optimal binding time for the inhibitors, the incubation time of the mixtures for the competitive binding assay were investigated and determined to be optimal at 2 h by detecting the concentration of the positive compound of SBC-115076 binding to PCSK9 in the absence of GST-EGF-A (Figure 3B). SBC-115076, a model inhibitor for PCSK9, was selected to verify the method established. As shown in Figure 3D, this method was demonstrated to be feasible to evaluate the effects of small molecules on the PCSK9/LDLR interaction.

### 2.3. Screening the Potential Natural Products Interrupting the PCSK9/LDLR Interaction

**Figure 4:**
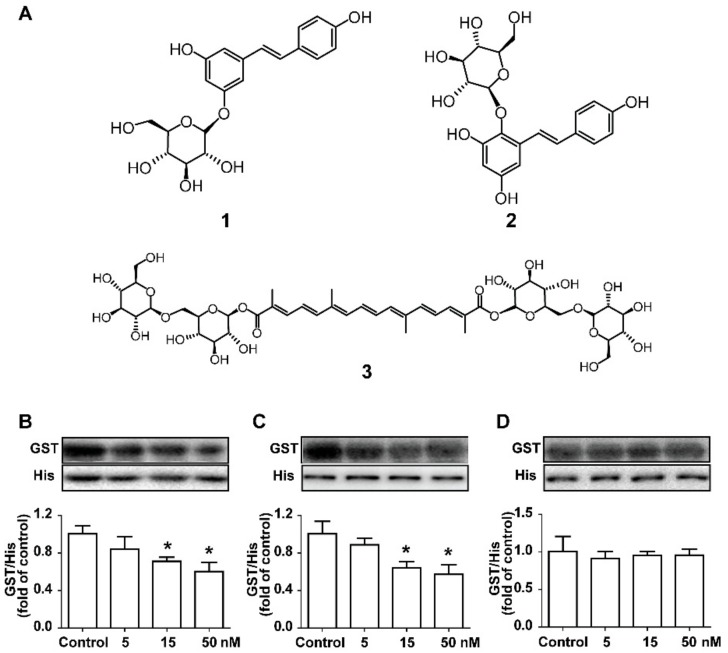
The potential inhibitory effect of the natural compounds on the PCSK9/LDLR interaction. (**A**) Chemical structures of compounds **1**–**3**, namely polydatin, tetrahydroxydiphenylethylene-2-*O*-glucoside and crocin I, respectively; (**B**–**D**) The potential inhibition of compounds **1**–**3** on the PCSK9/LDLR interaction were evaluated by the methods above. The GST/His ratios were monitored by western blot assay in the presence of compounds **1**–**3** at different concentrations (5, 15, and 50 nM), respectively. The values are the mean ± SEM deviation of the three independent experiments. * *p* < 0.05; ** *p* < 0.01, compared with the control group.

According to the method established above, we expected to explore the potential inhibition of natural products on the PCSK9/LDLR interaction. As examples, three famous natural active compounds with cholesterol-lowering effects, polydatin (**1**), tetrahydroxydiphenylethylene-2-*O*-glucoside (**2**) and crocin I (**3**), were selected for our screening research. The chemical structures of compounds **1**–**3** are shown in Figure 4A. Considering that the ratio of the inhibitors/PCSK9 was expected to be >1, the tested concentration ranges of the natural compounds were set according to the concentration of PCSK9 in the system. For compounds **1** and **2**, the GST/His ratios decreased in a concentration-dependent manner. When adding compounds **1** and **2** at the concentrations of 15 nM and 50 nM, the EGF-A binding to PCSK9 significantly decreased, leading us to conjecture that compound **1** and **2** had potential inhibitory effects on the PCSK9/LDLR interaction (Figure 4B,C). In contrast, compound **3** could not induce the decline of the GST/His ratios even at the highest concentration, representing weak inhibitory effects on the PCSK9/LDLR interaction (Figure 4D). According to the literature, compounds **1**–**3** had the potential for the development of lipid-lowering drugs [10,20,21,22]. However, only compound **1** was described in the previous literature for its interaction with PCSK9 [10]. This method presented the same results as previous studies of compound **1**, providing a new perspective for its direct effect on the PCSK9/LDLR interaction. For compounds **2** and **3**, this study was the first discussion of their effects on the PCSK9/LDLR interaction, and we presented a preliminary judgment on that issue. Although it was anticipated that our method could distinguish the inhibition of protein–protein interaction by the different natural compounds, the cell assays were essential.

### 2.4. The Potential Natural Inhibitors Prevent PCSK9-Mediated LDLR Degradation in HepG2 Cells

**Figure 5:**
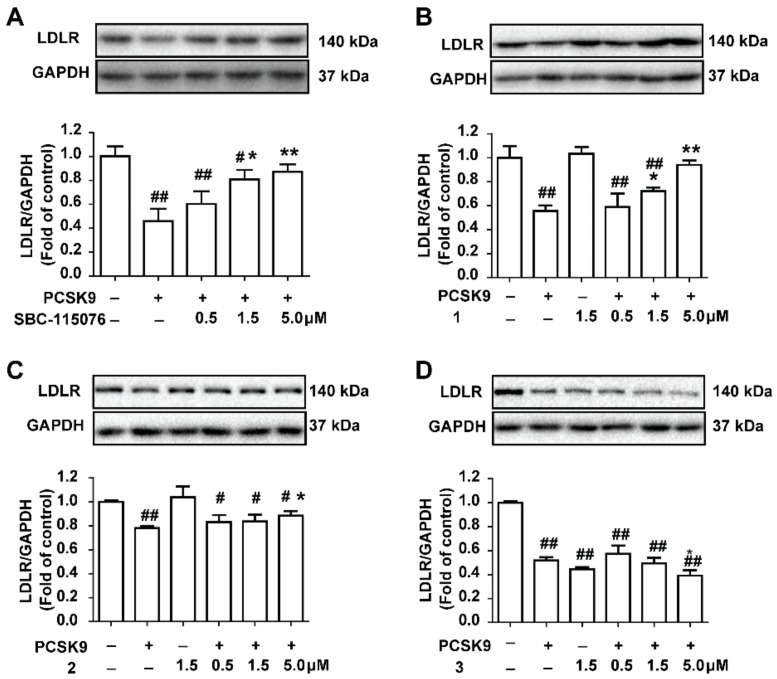
The activities of compounds **1**–**3** on preventing PCSK9-mediated LDLR degradation in the HepG2 cells. The LDLR levels in the HepG2 cells were monitored by western blot upon the treatment of PCSK9 in the presence of SBC-115076 (**A**); polydatin (**B**); tetrahydroxyl diphenylethylene-2-*O*-glucoside (**C**); and crocin I (**D**) at different concentrations (0, 0.5, 1.5, and 5.0 μM), respectively. The values are the mean ± SEM deviation of three independent experiments. ^#^ *p* < 0.05; ^##^ *p* < 0.01; compared with the control group, * *p* < 0.05; ** *p* < 0.01, compared with the PCSK9 group.

In order to illustrate the validity and practicability of the evaluation method above, a cell assay was performed for confirmation. The range of the tested concentrations of the compounds was set by referring to a previous study [16] to guarantee the feasibility. As shown in Figure 5A, SBC-115076 inhibited the PCSK9-mediated LDLR degradation in a concentration-dependent manner, which was the positive group. In the HepG2 cells, polydatin (**1**) and tetrahydroxydiphenylethylene-2-*O*-glucoside (**2**) inhibited the PCSK9-mediated LDLR degradation in a concentration-dependent manner (Figure 5), while crocin I (**3**) presented the opposite effects. For compound **1**, at the highest concentration tested (5 μM), the LDLR levels were restored to the control levels (Figure 5B). Similarly, compound **2** also restored the LDLR levels, but its activity was weaker than that of SBC-115076 and compound **1** (Figure 5C). In contrast, the results showed that compound **3** promoted the degradation of the LDLR, leading to lower levels of the LDLR than the model levels at the highest concentration tested (5 μM). To sum up, polydatin showed prominent inhibitory activities with respect to PCSK9-mediated LDLR degradation and could be considered to be a potential drug candidate. Polydatin is a major active component of *Polygonum cuspidatum* Sieb. et Zucc., possessing several pharmacological effects, such as lipid-lowering [20], anti-diabetes effects [10] and cardiovascular protection [27]. The interaction between polydatin and PCSK9 was demonstrated via molecular docking in previous reports [10]. Our study provided more evidence for its potential inhibition of the PCSK9/LDLR interaction directly, which might promote drug development using natural products. Tetrahydroxy- diphenylethylene-2-*O*-glucoside (**2**), a natural stilbene mainly distributed in *Polygonum multiflorum* Thunb, shows great activities in hepatic lipid regulation [28,29]. Previous studies have proven that compound **2** could inhibit the expression of the sterol regulatory element-binding protein 1c, and reduce the contents of acetyl-CoA carboxylase 1 and fatty acid synthase [21]. Our study provided new evidence of the lipid-lowering effect of compound **2**, which contributed to the understanding of the lipid-reducing mechanisms of stilbenes. Finally, it was shown that crocin I had little inhibition on the PCSK9/LDLR interaction according to this strategy, which reminded us that its lipid-reducing effect might be for other mechanisms.

## 3. Materials and Methods

### 3.1. Apparatus, Chemicals and Reagents

The standard of tetrahydroxydiphenylethylene-2-*O*-glucoside (**2**) was purchased from Chengdu Herbpurify Co., Ltd. (Chengdu, China). The standards of polydatin (**1**) and crocin I (**3**) were purchased from Shanghai Yuanye Bio-Technology Co., Ltd. (Shanghai, China). The purity of the standards was above >92% according to the normalization of the peak areas detected by HPLC. SBC-115076 was purchased from Selleck (Shanghai, China). All the other chemicals and solvents were of analytical grade. The plasmid isolation and DNA purification kits were provided by Transgene (Beijing, China). Isopropylthio-β-d-galactopyranoside (IPTG) was purchased from Invitrogen (Carlsbad, CA, USA). Ni-NTA affinity resin and GST-tag Purification Resin were purchased from Beyotime (Nanjing, China). Amicon Ultra Centrifugal Filter Units were purchased from Millipore (Bedford, MA, USA).

### 3.2. Construction, Expression, and Purification of His-PCSK9 Protein

The gene encoding residues 152-692 of human PCSK9 were codon-optimized for bacterial expression and synthesized commercially (Idobio, Nanjing, China). PCSK9 (152-692) was PCR-amplified from the synthesized gene and cloned into a pET22b vector between the NdeI and XhoI sites. It was designed to append a N-terminal fusion tag comprised of the hexahistidine binding protein. The plasmid was transformed into *Escherichia coli* BL21(DE3). The colonies were used to inoculate Terrific Broth (TB) media containing ampicillin (50 μg/mL) and were grown at 37 °C. In the mid-log phase, the recombinant protein expression was induced with the addition of IPTG to a final concentration of 0.1 mM. The cells were then left to grow overnight at 20 °C. All the purification steps were performed as rapidly as possible at 4 °C. The cells were pelleted by centrifugation, resuspended in 50 mM sodium phosphate (pH 8.0)/0.3 M NaCl and lysed through a microfluidizer. The lysate was clarified by centrifugation, and His-PCSK9 was purified by Ni-NTA resin column. The protein was step eluted with different concentrations of imidazole and the pooled fractions were concentrated by ultrafiltration to 0.5 mg/mL.

### 3.3. Construction, Expression, and Purification of GST-EGF-A Protein

The EGF-A of the LDLR were expressed as recombinant GST fusion proteins using the vector pGEX-6P-1 in *Escherichia coli* BL21(DE3) according to the literature [19]. The gene’s sequence of the EGF-A was listed as follows: GAATTCGGTA CCAACGAATG CCTGGACAAC AACGGTGGTT GCTCTCACGT TTGCAACGAC CTGAAAATCG GTTACGAATG CCTGTGCCCA GACGGCTTCC AGCTGGTTGC GCAGCGTCGT TGCGAATAAC TCGAG. The transformed cells were grown at 37 °C in a TB medium containing ampicillin (50 μg/mL) and induced with 0.2 mM IPTG to grow overnight at 20 °C. The cells were harvested by centrifugation at 6000 rpm for 5 min at 4 °C The cell pellets were resuspended in a lysate buffer (pH 7.3, containing 140 mM NaCl, 2.7 mM KCl, 10 mM Na_2_HPO_4_, and 1.8 mM KH_2_PO_4_) and lysed through a microfluidizer. The lysate was clarified by centrifugation and purified by GST-tag purification resin column. After washing with a lysate buffer, the protein was step eluted with different column volumes of elution buffer (pH 8.0, containing 50 mM Tris-HCl and 10 mM GSH) and the pooled fractions were concentrated by ultrafiltration to 4.0 mg/mL.

### 3.4. Interaction between PCSK9 and EGF-A by Fishing with Magnetic Beads

The Ni^2+^ of the Ni magnetic beads (MBs) and the hexahistidine tag of PCSK9 were linked by chelate action. An aliquot of 100 μL of the MBs (~10 mg) was transferred to a 1.5 mL centrifuge tube and washed with 100 μL of wash buffer (20 mM sodium phosphate, 500 mM NaCl, pH 7.4) three times. Then, 1 mL of the PCSK9 solution (50 μg/mL) was added to the magnetic beads. The mixture was shaken and rotated in a stirring mixer. The immobilization time (15, 30, 60, and 120 min) was investigated to optimize the preparation conditions. After incubation, the supernatant was discarded. Then, 100 μL of wash buffer (20 mM sodium phosphate, 500 mM NaCl, pH 7.4) was added to exclude non-specific adsorption, and the PCSK9-coated magnetic beads (PCSK9-MBs) were prepared. The PCSK9 immobilized on the MBs were eluted by elution buffers (20 mM sodium phosphate, 500 mM NaCl, 400 mM imidazole, pH 7.4) three times with 200 μL, 100 μL, and 100 μL, respectively. The concentration of the eluted PCSK9 was measured by bicinchoninic acid (BCA) protein assay for calculating the concentration of PCSK9 on the MBs.

Different concentrations of GST-EGF-A (1.6, 2.4 and 4.0 μg/μL magnetic beads) were mixed with the PCSK9-MBs to ensure that there were sufficient amounts of the EGF-A for binding the PCSK9-MBs. After washing with 100 μL of wash buffer three times, the elution buffers (20 mM sodium phosphate, 500 mM NaCl, 400 mM imidazole, pH 7.4) were added three times in the amounts of 200 μL, 100 μL and 100 μL, respectively. The eluents were put together to collect the PCSK9/EGF-A complexes for further detection by western blot analysis.

### 3.5. Evaluation of Inhibitory Activities on PCSK9/LDLR Interaction

SBC-115076 was selected as the model inhibitor to verify the feasibility of the method [30], PCSK9-MBs were prepared as described above. An aliquot of 100 μL PCSK9-MBs was mixed with SBC-115076 (5 μM, 15 μM and 50 μM) and GST-EGF-A (2.4 μg/μL PCSK9-MBs) in PBS (75 mM, pH 7.4) for a total volume of 1 mL. The mixtures were shaken and rotated for a period to allow the ligands to bind to PCSK9. The incubation time (0.5, 1, 2, 4 and 8 h) was optimized by detecting the concentration of SBC-115076 bonded to the PCSK9-MBs in the absence of GST-EGF-A. SBC-115076 that were bonded to PCSK9 were dissociated from PCSK9 by adding equivoluminal methanol and were analyzed by HPLC. When the competitive incubation was completed, the non-specific adsorption was washed by PBS, and then, the PCSK9/EGF-A or PCSK9/compounds complexes could be eluted by imidazole and prepared for western blot assay. The His-tags and GST-tags were detected respectively and the GST/His ratio was considered to be a feature of the inhibitory activities. Natural products including polydatin, tetrahydroxydiphenylethylene-2-*O*-glucoside and crocin I, were evaluated for their inhibitory activities on the PCSK9/LDLR interaction according to the method described above.

### 3.6. HPLC Analysis for SBC-115076

Chromatographic analysis was performed on an Agilent 1260 series HPLC system (Agilent Technologies, Santa Clara, CA, USA) equipped with a binary pump, an online degasser, an auto plate-sampler, a thermostatically controlled column compartment and an ultraviolet (UV) detector. The sample separation was achieved on an Agilent ZORBAX SB-C18 column (4.6 mm × 250 mm, 5 µm) with a constant flow rate of 0.8 mL/min at 35 °C. The mobile phase was composed of water (0.1% formic acid) (A) and acetonitrile (B) using isocratic elution at 30% B. The sample volume injected was set at 40 µL. The peaks were monitored at 325 nm with a reference at 550 nm.

### 3.7. Cell Culture and Measurement of LDLR in HepG2 Cells

The HepG2 cells (American Type Culture Collection, Manassas, VI, USA) were grown at 37 °C in Dulbecco’s Modified Eagle Medium (DMEM) (Sigma, Santa Clara, CA, USA) containing: 10% (*v*/*v*) fatal bovine serun (FBS) (Sigma, USA), penicillin (50 U/mL), streptomycin (50 g/mL) and L-glutamine (2 mM). 

The cells were grown in a humidified atmosphere of 95% air/5% CO_2_ at 37 °C, and in 6 multi-well plates at the proper cell densities. The HepG2 cells were incubated for 12 h and then divided into different groups for different treatments. The compounds at various concentrations were preincubated with 15 μg/mL PCSK9 in the presence of 0.5% DMSO for 30 min prior to the addition to the cells [16]. After 24 h incubation, the cells were measured for LDLR levels by western blot analysis. All the experiments were performed in triplicate.

### 3.8. Western Blot Analysis

The lysis buffer was prepared by adding protease and phosphatase inhibitor cocktail (100×; Thermo, Waltham, MA, USA) to a RIPA lysis buffer (pH 8.0, 50 mM Tris, 150 mM NaCl, 0.02% sodium azide, 0.1% SDS, 1% NP-40, 0.5% sodium deoxycholate, 1 mM EDTA, and so on). For the cells, the plate was rinsed with an ice-cold PBS buffer three times, and then, the cells were lysed for 30 min. The lysates or homogenates were centrifuged, and then, the supernatant was collected for analysis by adding a 5 × SDS loading buffer containing 7% β-mercaptoethanol. For the elution solution containing the PCSK9/LDLR complexes, the supernatant was boiled with the addition of a 5 × SDS loading buffer containing 7% β-mercaptoethanol directly. Equal amounts of the protein samples were separated by 8% (*v*/*v*) SDS-PAGE and electrophoretically transferred onto a PVDF (0.22 μm, Millipore, Burlington, MA, USA). After blocking with 5% BSA-Tris buffered saline containing 0.1% Tween 20 at room temperature for 2 h, the membranes were incubated with the corresponding primary anti-LDLR (1:4000; Abcam, Cambridge, UK), anti-GAPDH (1:4000; Sigma), anti-6×His tag (1:4000; Yeasen, Shanghai, China) and anti-GST tag (1:4000; Yeasen) antibodies at 4 °C overnight. The membranes were then incubated with HRP-conjugated anti-rabbit IgG (1:4000; Cell Signaling Technology, Danvers, MA, USA) and anti-mouse (1:4000; Cell Signaling Technology) secondary antibodies for 2 h at room temperature, and the immunoreactive protein bands were visualized using enhanced chemiluminescence reagents (Bio-Rad, Hercules, CA, USA). The intensity of the protein bands was quantified using the Image Lab analysis software.

## 4. Conclusions

In conclusion, we proposed a composite strategy to evaluate the effects of natural products on preventing the interaction of PCSK9/LDLR directly. Based on the proposed strategy, two natural compounds, polydatin (**1**) and tetrahydroxydiphenylethylene-2-*O*-glucoside (**2**), were successfully identified as inhibitors for the PCSK9/LDLR interaction and were proven to prevent PCSK9-mediated LDLR degradation in the HepG2 cells. Thus, this strategy was considered to be accurate and convenient for discovering potential new candidates to inhibit the protein–protein interaction. This study is expected to be used as a preliminary screening method to further discover new drug candidates and provide a new perspective for the treatment of PCSK9-mediated hypercholesterolemia.
